# Protective effect of remote liver ischemic postconditioning on pulmonary ischemia and reperfusion injury in diabetic and non-diabetic rats

**DOI:** 10.1371/journal.pone.0268571

**Published:** 2022-05-26

**Authors:** Dou Huang, Changwei Chen, Yunxia Zuo, Lei Du, Ting Liu, Geoffrey W. Abbott, Zhaoyang Hu

**Affiliations:** 1 Department of Anesthesiology, West China Hospital, Sichuan University, Chengdu, Sichuan, China; 2 Laboratory of Anesthesia and Critical Care Medicine, National-Local Joint Engineering Research Centre of Translational Medicine of Anesthesiology, West China Hospital, Sichuan University, Chengdu, Sichuan, China; 3 Bioelectricity Laboratory, Department of Physiology and Biophysics, School of Medicine, University of California, Irvine, CA, United States of America; Thomas Jefferson University, UNITED STATES

## Abstract

Pulmonary ischemia and reperfusion (I/R) injury occurs in many clinical conditions and causes severe damage to the lungs. Diabetes mellitus (DM) predisposes to pulmonary I/R injury. We previously found that remote liver ischemia preconditioning protected lungs against pulmonary I/R injury. The aim of the present study was to investigate whether remote liver ischemic postconditioning (RLIPost) attenuates pulmonary damage induced by I/R injury in non-diabetic or diabetic rats. Male Sprague-Dawley rats were assigned into non-diabetic and diabetic groups. All rats except for the sham were exposed to 45 min of left hilum occlusion followed by 2 h of reperfusion. RLIPost was conducted at the onset of pulmonary reperfusion by four cycles of 5 min of liver ischemia and reperfusion. Lung injury was assessed by the wet/dry weight ratio, pulmonary oxygenation, histopathological changes, apoptosis and the expression of inflammatory cytokines. Reperfusion-associated protein phosphorylation states were determined. RLIPost offered strong pulmonary-protection in both non-diabetic and diabetic rats, as reflected in reduced water content and pulmonary structural damage, recovery of lung function, inhibition of apoptosis and inflammation after ischemia-reperfusion. RLIPost induced the activation of pulmonary STAT-3, a key component in the SAFE pathway, but not activation of the proteins in the RISK pathway, in non-diabetic rats. In contrast, RLIPost-induced pulmonary protection in diabetic lungs was independent of SAFE or RISK pathway activation. These results demonstrate that RLIPost exerts pulmonary protection against I/R-induced lung injury in non-diabetic and diabetic rats. The underlying mechanism for protection may be different in non-diabetic (STAT-3 dependent) versus diabetic (STAT-3 independent) rats.

## Introduction

Lung ischemia/reperfusion (I/R) injury is characterized by alveolar damage, interstitial edema, hypoxemia and inflammatory cell infiltration occurring following heart or lung transplantation, cardiopulmonary bypass surgery or pulmonary thrombolysis [1]. Specifically, pulmonary I/R injury may lead to primary graft dysfunction (PGD), which is the dominant cause of early morbidity and mortality after lung transplantation [2]. Therefore, it is urgent to identify effective therapeutic approaches to alleviate lung damage after lung I/R injury and improve survival.

The concept of ischemic preconditioning (IPC), introduced by Murry *et al* in 1986., is that intermittent brief episodes of short ischemic stimuli may render the heart more resistant to subsequent sustained ischemic insults [3]. Moreover, IPC applied in distant tissues or organs can also protect hearts, known as remote ischemic preconditioning (RIPC) [4]. We [5,6] and others [7] previously found that RIPC (induced in livers or limbs) exerted strong cardioprotective effects against myocardial I/R injury in rodent animals. Importantly, although hearts may serve as the most common target for RIPC, remote limb ischemic preconditioning was shown to preserve lung function after acute lung injury in a porcine model [8], and to reduce lung damage after an open heart operation in infants [9]. Furthermore, this brief remote ischemic preconditioning stimuli conducted in the liver, the largest metabolic organ, raised the tolerance to reperfusion-induced pulmonary damage, and further inhibition of pulmonary STAT3 phosphorylation was responsible for the underlying mechanism of RLIPC-associated lung protection [10]. However, because of the unpredictability of acute ischemic syndromes, remote ischemic conditioning performed during the reperfusion period (remote ischemic postconditioning, RIPost) acts as a more promising therapeutic strategy and several studies have demonstrated the efficacy of remote limb postconditioning in patients [11] or animal models [12] with ischemia and reperfusion. Based on these studies, which serve as a foundation for potential RIPost-targeted therapies in I/R injury, we hypothesized that remote liver postconditioning might induce pulmonary protection in the setting of lung I/R injury.

Diabetes mellitus (DM) is a growing epidemic associated with multiple complications. Hyperglycemia affects the microvascular function in the respiratory system and thus the lung is considered as a target organ in DM. It has been shown that diabetes can exacerbate I/R injury [13,14]. Although the beneficial effects of ischemic conditioning have been fully confirmed, preclinical studies have demonstrated that the presence of diabetes can limit the protective effects offered by ischemic conditioning [15]. Therefore, in our study, we also aimed to study the pulmonary protective role of remote liver postconditioning in STZ-induced diabetic rats.

Taken together, our aims here were to determine whether remote liver conditioning performed at the onset of pulmonary reperfusion preserves non-diabetic or diabetic lung function and reduces pulmonary damage after lung I/R injury, and further clarify the potential underlying mechanism for any observed beneficial effects.

## Materials and methods

### Ethical approval

The current investigations were performed using a protocol approved by the Institutional Animal Care and Use Committee of Sichuan University (Sichuan, China, Permit Number: 20211396A) and all the procedures and protocols were done according to the recommendations in the Guide for the Care and Use of Laboratory Animals of the National Institutes of Health (8th edition, 2011).

### Animals

Adult male Sprague-Dawley rats (220-250 g) of 7–8 weeks of age were used and were housed in a room with controlled temperature (20–25°C) and humidity (60 ± 5%) under a 12-h light–dark cycle. All rats were fed standard pellet diet and water *ad libitum*.

### Study design

**Fig 1:**
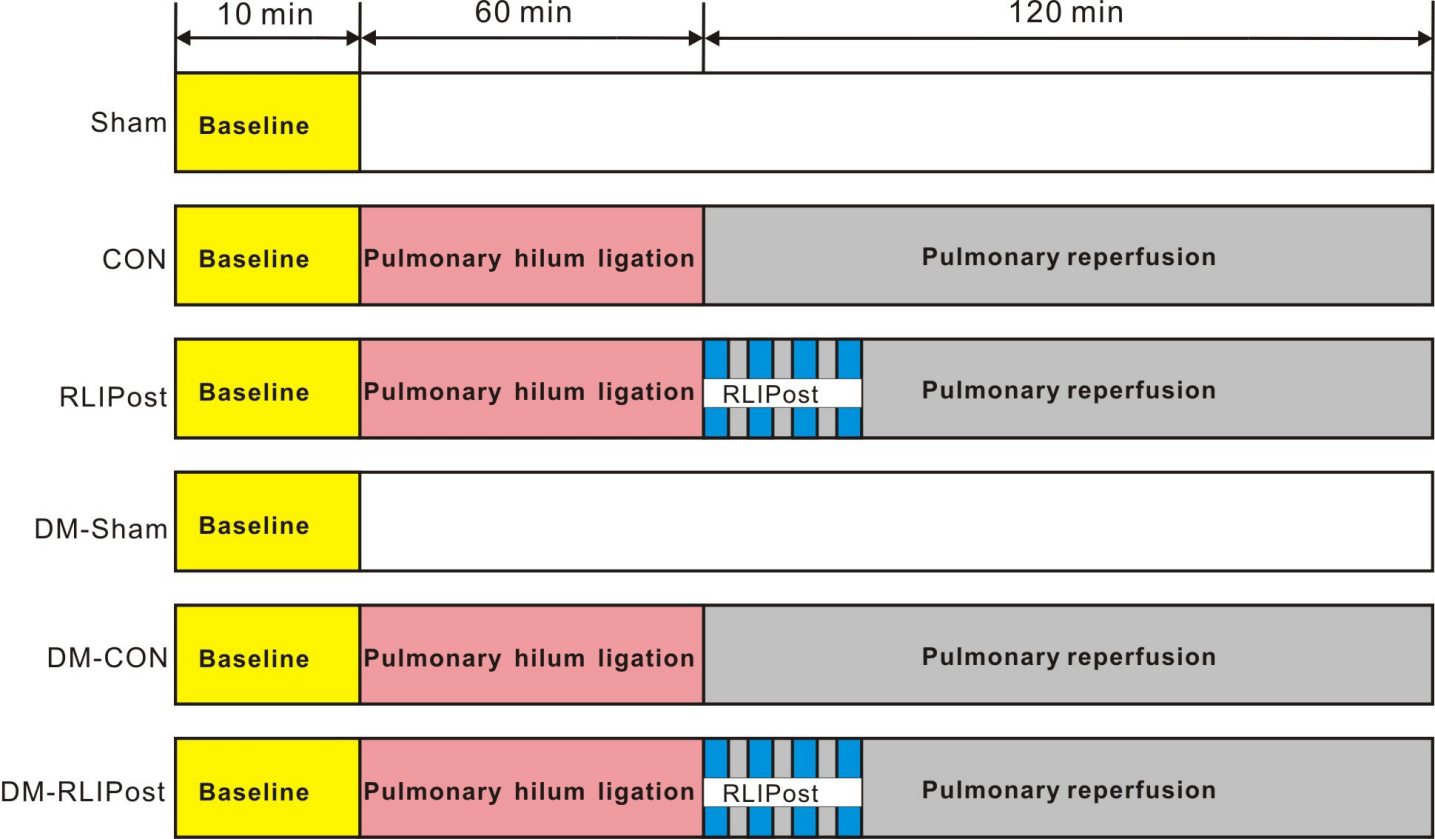
Experimental protocols. Lungs except for the sham-operated ones were subjected to pulmonary ischemia/reperfusion injury by ligation of the left pulmonary hilus for 60 min, followed by 120 minutes of reperfusion. Remote liver ischemic postconditioning (RLIPost) was conducted at the onset of pulmonary reperfusion by four cycles of 5 min of liver ischemia with 5 min intermittent reperfusions. Sham: sham-operated, CON: control, RLIPost: remote liver ischemia postconditioning, DM: STZ-induced diabetes.

The experiment protocol is illustrated in ***Fig 1***. Rats were randomly assigned to the following groups: (1) Nondiabetic sham group (sham): the laparotomy was performed, and the liver hilum was exposed without further intervention. The thoracotomy was performed, and the left lung hilum was identified but was not occluded; (2) Nondiabetic control group (CON): the laparotomy was performed without further hepatic intervention. The left pulmonary hilum was completely occluded using a microvascular clamp; (3) Nondiabetic remote liver ischemic postconditioning group (RLIPost): conditioning was exerted at the onset of pulmonary reperfusion by four cycles of 5 min of liver ischemia and subsequent 5 min of reperfusions; (4) STZ-induced diabetic sham group (DM-sham): STZ was used to induce diabetes, rats received the same surgical manipulation without liver or lung clamping; (5) STZ-induced diabetic control group (DM-CON):diabetic rats underwent 60 min of left hilum occlusion followed by 120 min of reperfusion; (6) STZ-induced diabetic remote liver ischemic postconditioning group (DM-RLIPost): diabetic rats had four cycles of liver postconditioning treatment at the onset of pulmonary reperfusion.

### Induction of pulmonary I/R injury

As we previously described [10,16], rats were anesthetized using sodium pentobarbital (50 mg/kg, intraperitoneal injection). Adequacy of anesthesia was controlled by monitoring heart rates, corneal reflex, respiration, and the lack of response to toe-pinching. After stabilization, a tracheotomy was performed. The trachea was intubated with a 14-gauge catheter (B. Braun Melsungen AG, Melsungen, Germany) and the lungs were ventilated with a rodent ventilator (Chengdu Taimeng Technology Co., Ltd., Chengdu, Sichuan, China). The initial tidal volume was 8–10 ml/kg, the expiration:inspiration ratio was 5:4, and the frequency was 60–70 times per minute. Following a 2-cm left anterolateral thoracotomy, the left lung hilum was identified and occluded with a small microvascular clamp. During the 60 min of pulmonary ischemia period, the tidal volume was 6 ml, expiration:inspiration was 5:4 and the frequency was 70–80 times per minute. At the end of pulmonary ischemia, the clamp was released, and reperfusion was conducted for 120 minutes. Lungs were kept moist with warm saline-soaked gauze. During the entire reperfusion period, the tidal volume, expiration:inspiration ratio and the frequency were set back to 8–10 ml/kg, 5:4 and 60–70 times per minute, respectively. A warming blanket was placed underneath each rat throughout the experiment. The left pulmonary hilum occlusion was not performed in sham-operated rats. At the end of the reperfusion, rats were euthanized using excessive anesthesia administration (sodium pentobarbital, 200 mg/kg, i.p.).

### Lung wet-to-dry weight ratio

At the end of the experiment, the lower lobe of the left lung was dissected and weighed (Wet weight). The lung was then incubated at 60°C for 72 h and weighed (Dry weight). The wet/dry weight ratio (wet weight-dry weight) / wet weight x100) was used as an index of lung water content.

### Blood gas measurement

The femoral artery was cannulated via a 24-G heparin-filled catheter (B. Braun Melsungen AG, Melsungen, Germany) for arterial blood collection. Arterial blood (0.2 mL) was collected from each rat at baseline as well as 1 h and 2 h after reperfusion. Blood gas was measured using a blood gas analyzer (ABL800 FLEX, Radiometer Medical A/S, Copenhagen, Denmark). Oxygenation index is the ratio of arterial oxygen partial pressure to fractional inspired oxygen.

### Lung tissue collection

In a parallel study, at the end of the experiment, the lower lobe of the left lung was collected and fixed with 10% formaldehyde overnight. The paraffin wax-embedded lung tissues were then cut into 4 μm sections and mounted on glass slides.

### Histologic analysis

After deparaffinization and rehydration, lung tissue sections were stained with hematoxylin-eosin (H&E) and were ranked by two independent researchers who were blind to the experimental grouping. The pathologic changes included alveolar edema, alveolar septal thickening, alveolar and interstitial hemorrhage and inflammatory infiltration. These pathologic changes were evaluated based on a 0–2 scale scoring system as described previously [10,16,17]. Ten different fields were chosen from each slice at random.

### TUNEL assay

Terminal deoxynucleotidyl transferase-dUTP nick-end labeling (TUNEL) staining was performed according to the manufacturer’s instructions (DeadEnd™ Fluorometric TUNEL system, Promega, Madison, WI, USA). Ten visual fields were randomly selected from each slice and analyzed blindly by two independent researchers using an upright microscope (Eclipse Ni-E, Nikon, Tokyo, Japan). TUNEL-positive cells were stained green and DAPI was used as a marker for the cell nucleus (stained blue). The apoptotic index was characterized as the percentage of TUNEL-positive apoptotic nuclei to total DAPI-positive nuclei.

### Immunohistochemical staining of IL-6 and TNF-α

Immunohistochemical staining was performed for tumor necrosis factor alpha (TNF-α) and interleukin 6 (IL-6). The lung slices were deparaffinized and then underwent antigen retrieval at 100°C for 15 min before being incubated in a 1% hydrogen peroxide solution for 20 min. The sections were then washed and treated with a goat serum blocking solution for 40 min at 37°C, and subsequently incubated with primary antibody raised against IL-6 and TNF-α (all 1:200, Affinity bioscience, Cincinnati, OH, USA) overnight at 4°C. All tissue sections were rinsed in distilled water and were then incubated with secondary antibody for 30 min at 37°C. The sections were further reacted with 3,3’-diaminobenzadine (DAB) and were counterstained with hematoxylin. Negative control slices were treated with phosphate-buffered saline in lieu of the primary antibody. Images were captured by an inverted microscope (CAST system, Olympus A/S, Ballerup, Denmark). Ten fields were randomly selected from each slice and analyzed in a blinded manner. Cells with yellow/brown staining of IL-6 or TNF-α in the cytoplasm were considered as positive expression. Images were analyzed using Image-pro plus software (Media Cybernetics Inc., Carlsbad, CA, USA).

### Western blotting

The left lung tissue was homogenized and sonicated in a tissue lysis buffer containing 1 mM EDTA, 150 mM NaCl, 50 mM Tris-HCl (pH7.4), 1% NP-40, 0.25% sodium deoxycholate, 1X protease inhibitor cocktail (Sigma- Aldrich, St. Louis, MO, USA) and 1X protease inhibitor cocktail (Sigma- Aldrich, St. Louis, MO, USA). The homogenate was then centrifuged at 10,000 g at 4°C for 10 min. The supernatant was collected as a total protein preparation and the bicinchoninic acid (BCA) protein assay kit (Pierce, Rockford, IL, USA) was used to determine the protein concentration. Equal amounts of lung homogenates were loaded and separated by 12% sodium dodecyl sulfate-polyacrylamide gel electrophoresis. The protein was then transferred to a nitrocellulose blotting Membrane (Pall Corporation, Pensacola, USA). The membranes were incubated with 5% non-fat milk in PBS buffer containing 0.1% Tween-20 (PBST) for 1 hour at room temperature and then incubated (overnight, 4°C) with primary antibodies including extracellular signal-regulated kinase1/2 (ERK1/2)(Thr202/Tyr204)(p-ERK), total ERK1/2, phosphorylated glycogen synthase kinase-3β(Ser9) (p-GSK-3β), total GSK-3β, phosphorylated Akt (ser473)(p-AKT), total Akt, phosphorylated P38 MAPK (Thr180/Tyr182) (p-P38 MAPK), total P38 MAPK, phosphorylated STAT-5 (Tyr694)(p-STAT5) and phosphorylated STAT-3 (Tyr705)(p-STAT3) and total STAT-3. After being washed with PBST, the membranes were incubated with horseradish peroxidase-conjugated goat-anti-rabbit IgG antibody (1:5000, Bio-Rad, Hercules, CA, USA) for 2 hours at room temperature. With an enhanced chemiluminescence detection system (GE Healthcare, Rahway, NJ, USA), proteins were visualized using an AmershamImager 600 system (GE Healthcare, Little Chalfont, UK). The band densities were determined by ImageJ Software (National Institutes of Health, Bethesda, MD, USA). The relative abundance was obtained by normalizing the density of the phosphorylated protein against that of total protein.

### Statistical analysis

All data are presented as the mean ± standard deviation. The experimental results were analyzed by SPSS 16.0 software (SPSS, Inc., Chicago, IL, USA) or Graphpad Prism 8 software (GraphPad, La Jolla, CA, USA). The differences among groups over three were assessed by one-way analysis of variance. Levene’s test was used to test homogeneity of variance. When statistically significant differences were detected, the Student-Newman-Keuls test was performed to identify differences when the variance was equal, or Dunnett’s T3 test was used when equal variances were not assumed. The statistical test for PaO_2_ changes over time was performed by two-way repeated-measures analysis of variance (two-way ANOVA). The interactions between group and time (group x time interaction) were tested. In this test, group was a Between-Subjects factor and time was a Within-Subjects factor. If significance was detected in the test of Between Subjects, the differences among groups were assessed by one-way analysis of variance with the Bonferroni test for variances. Statistical significance was defined as P less than 0.05.

## Results

### RLIPost decreased I/R-induced pulmonary edema

**Fig 2:**
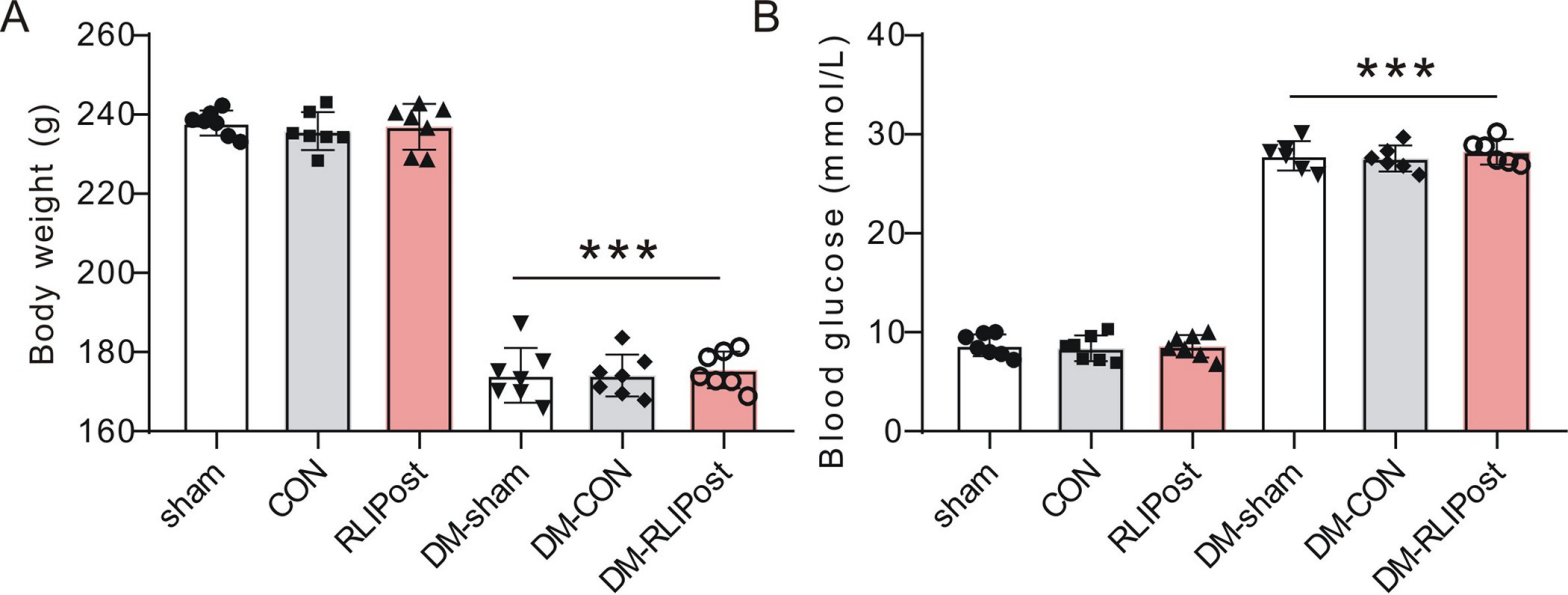
The effect of STZ on body weight and plasma glucose. A. The effect of STZ treatment on body weight. Values are mean ± SD. ***P<0.001, compared with nondiabetic rats. Sham: sham-operated, CON: control, RLIPost: remote liver ischemia postconditioning. DM: STZ-induced diabetes. n = 7 per group. B. The effect of STZ treatment on plasma glucose. Values are mean ± SD. ***P<0.001, compared with nondiabetic rats. n = 6–7 per group.

**Fig 3:**
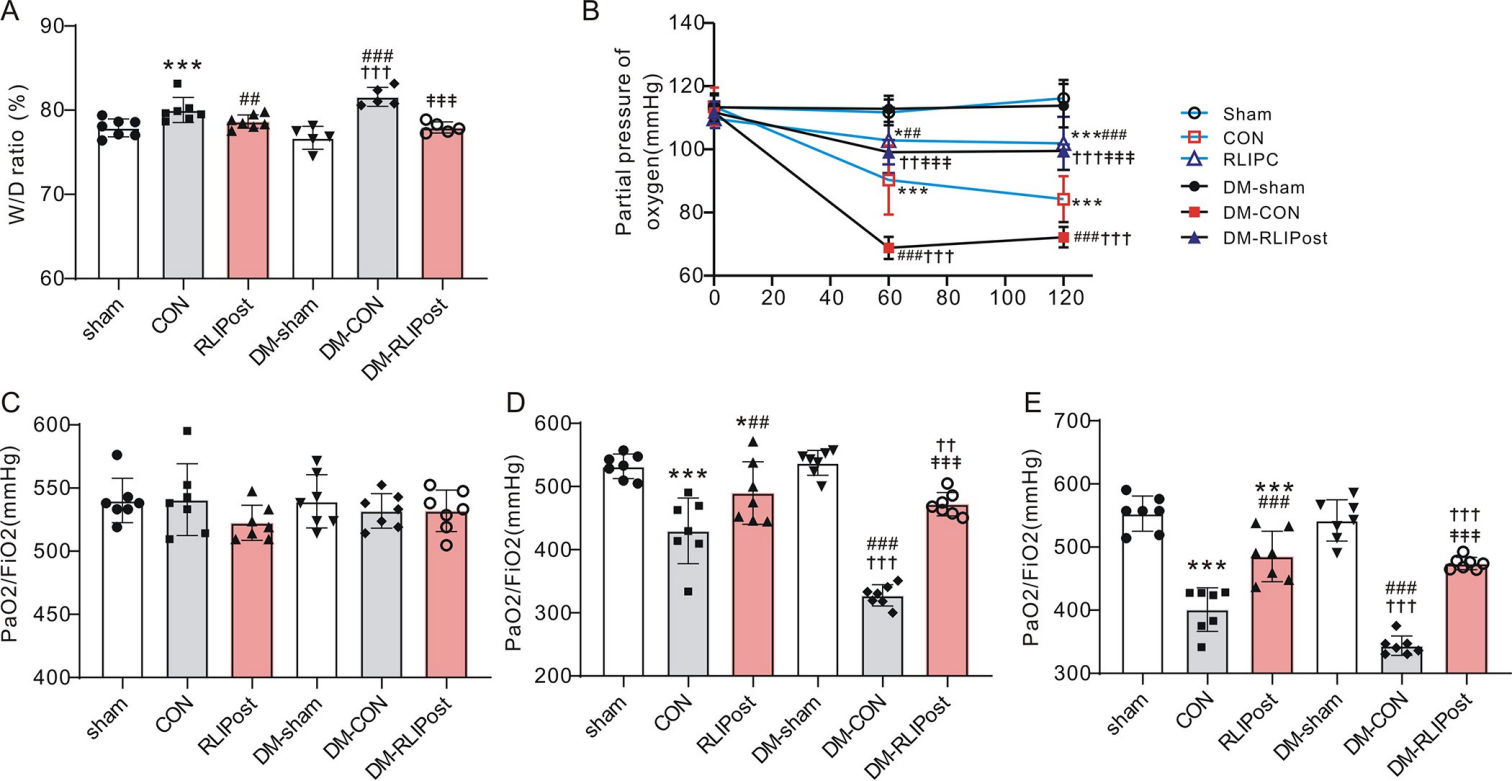
RLIPost ameliorates ischemia reperfusion-induced lung injury. A. Ratio of wet/dry weight in lung tissues. The data are presented as the mean ± standard deviation. ***P<0.001 vs. the sham lungs. ^##^: P<0.01, ^###^: P<0.001, vs. CON. ^†††^: P<0.001, vs. DM-sham. ^‡‡‡^: P<0.001 vs. DM-CON. n = 7 per group. sham: sham-operated, CON: control, RLIPost: remote liver ischemia postconditioning. DM: STZ-induced diabetes. B. Changes in partial pressure of oxygen. n = 7 in each group. *P<0.05, ***P<0.001, versus the sham group, ^##^P<0.01, ^###^P<0.001 versus the CON group, ^††^P<0.01, ^†††^P<0.001 versus the DM-sham group, ^‡‡‡^: P<0.001 vs. DM-CON. C. Changes in oxygenation index at baseline. n = 7 in each group. D. Changes in oxygenation index at 60 minutes after reperfusion. n = 7 in each group. *P<0.05, ***P<0.001, versus the sham group, ^##^P<0.01, ^###^P<0.001 versus the CON group, ^††^P<0.01, ^†††^P<0.001 versus the DM-sham group, ^‡‡‡^: P<0.001 vs. DM-CON. E. Changes in oxygenation index at 120 minutes after reperfusion from sham-operated, CON and RLIPost rats; n = 7 in each group. ***P<0.001, versus the sham group, ^###^P<0.001 versus the CON group, ^†††^P<0.001 versus DM-sham, ^‡‡‡^: P<0.001 vs. DM-CON.

Acute Type 1 diabetes mellitus (DM) was induced by the injection of streptozotocin. After 7 days of injection, STZ-treated rats exhibited diabetic profiles with decreased body weights (P<0.001, ***Fig 2A***) and higher levels of serum glucose (P<0.001, ***Fig 2B***) as compared to non-STZ-treated rats. Pulmonary edema was assessed by Wet-to-Dry (W/D) ratios. For non-diabetic rats, the W/D ratios in the control lungs (80.1±1.5%) were significantly higher than those in the sham-operated (77.9±1.1%, P<0.01) or RLIPost (78.7±0.80%, P<0.05) lungs. However, The W/D ratios in the RLIPost group were equivalent to those in the sham-operated group (P>0.05), indicating that RLIPost treatment exerted protection against I/R induced pulmonary edema in non-diabetic rats (***Fig 3A***). Next, we further checked the therapeutic efficacy of RLIPost in diabetic lungs. After reperfusion, control rats with STZ treatment (81.6±1.1%) had markedly higher W/D ratio than did sham diabetic (76.7±1.4%, P<0.001) or RLIPost-treated diabetic rats (78.0±0.7%, P<0.001). However, RLIPost treatment significantly ameliorated pulmonary edema when compared with DM-CON group (P<0.001, ***Fig 3A***). Taken together, these results suggested that RLIPost exerts pulmonary protection in both non-diabetic and diabetic rats after reperfusion.

### RLIPost improved pulmonary function post I/R

The beneficial effects of remote liver ischemic postconditioning on lung function as measured by PaO_2_ and oxygenation index (PaO_2_/FiO_2_) are shown in ***Fig 3B–3E.*** Under baseline conditions, the values of PaO_2_ and PaO_2_/FiO_2_ were similar among all rat groups in the presence or absence of diabetes (P>0.05). Repeated two-way ANOVA showed statistically significant differences among diabetic and non-diabetic sham, CON and RLIPost groups in PaO_2_ and PaO_2_/FiO_2_ over the period of measurement (P<0.0001) and a significant group-by-time interaction was detected among groups (P<0.0001). After reperfusion, PaO_2_ and PaO_2_/FiO_2_ decreased significantly in the non-diabetic and diabetic control groups when compared to their respective baseline (P<0.001) and sham-operated groups (P<0.001). However, the RLIPost-treated non-diabetic rats had significantly higher PaO_2_ and PaO_2_/FiO_2_ levels at 60 min (P<0.01 vs. CON) or at 120 min (P<0.001 vs. CON) post-I/R. Meanwhile, at 60 and 120 min of reperfusion, PaO_2_ and oxygenation index decreased in the DM-CON group (P*<*0.001 vs. DM-sham). However, RLIPost was highly effective in improving PaO_2_ and oxygenation index in diabetic lungs during the entire reperfusion period when compared with DM-CON rats (P<0.001).

### Pulmonary expression of proinflammatory cytokines

**Fig 4:**
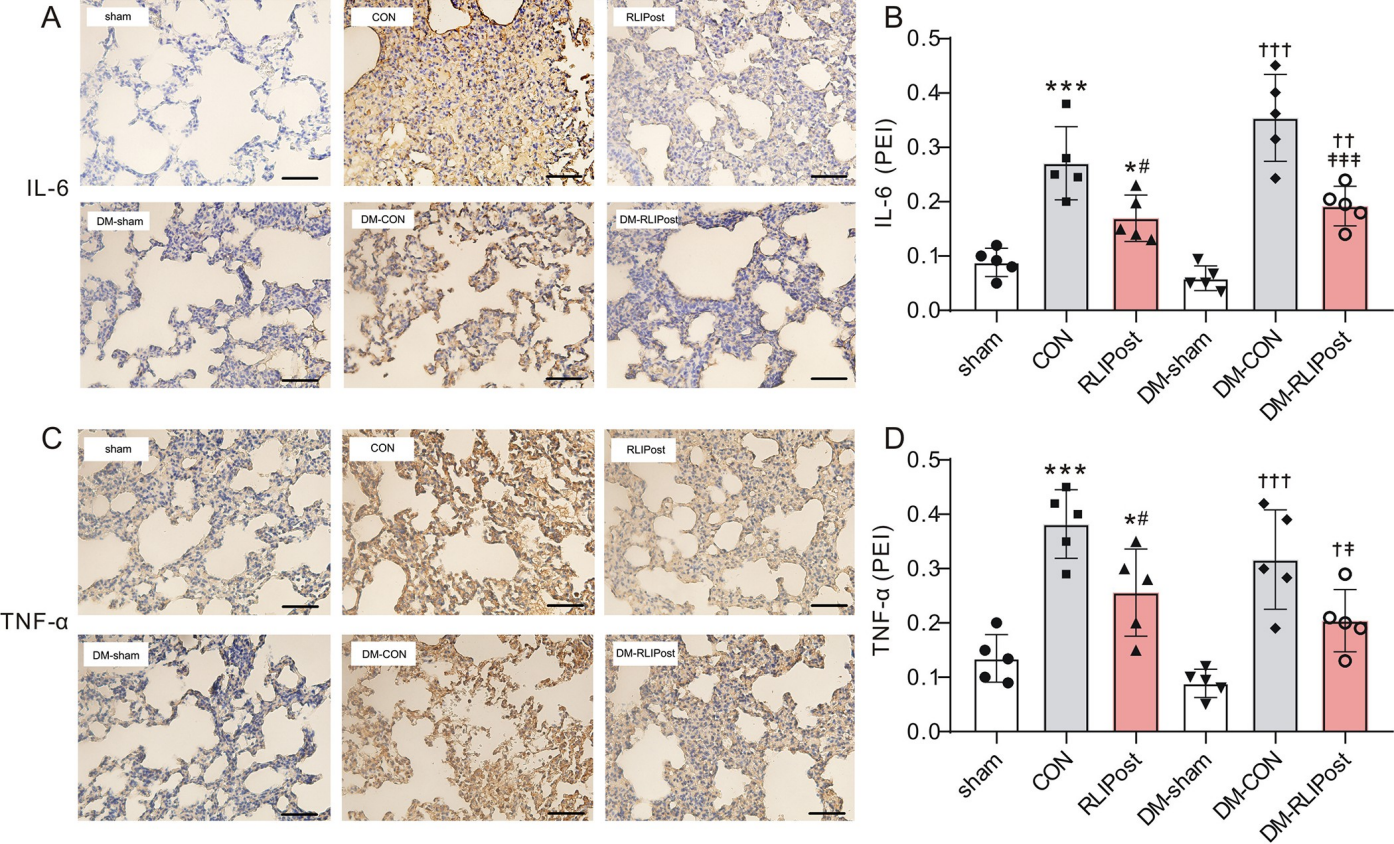
Effects of RLIPost on the expression of inflammatory cytokine. A. Representative immunostainings of IL-6 protein in the lung tissue sections obtained after 2h of reperfusion. sham: sham-operated, CON: control, RLIPost: remote liver ischemia postconditioning, DM: STZ-induced diabetes. scale bars, 50 μm. B. Densitometric analysis of IL-6 protein expression. n = 5 in each group. Values are mean ± SD. *P<0.05, ***P<0.001, versus the sham group, ^#^P<0.05, versus the CON group, ^††^P<0.01, ^†††^P<0.001 versus the DM-sham group, ^‡‡‡^: P<0.001 vs. DM-CON. C. Representative immunostainings of TNF-α protein in the lung tissue sections obtained after 2h of reperfusion. D. Densitometric analysis of TNF-αprotein expression. n = 5 in each group. Values are mean ± SD. *P<0.05, ***P<0.001, versus the sham group, ^#^P<0.05, versus the CON group, ^†^P<0.05, ^†††^P<0.001 versus the DM-sham group, ^‡^: P<0.05 vs. DM-CON.

Meanwhile, the effect of RLIPost on the production and release of proinflammatory cytokines, including IL-6 and TNF-α, was examined using immunohistochemistry on lung sections. As shown in ***Fig 4A–4D***, for non-diabetic lungs, we observed very low levels of IL-6 and TNF-α in the sham-operated lungs, however, the expression levels of these two cytokines were significantly elevated in the CON group as compared with the sham-operated group (P<0.001), indicative of significant lung damage with an inflammatory response after I/R. Notably, RLIPost markedly decreased the expression of IL-6 (P<0.05 vs. CON) and TNF-α (P<0.05 vs. CON) after reperfusion. Also, as expected, I/R injury significantly increased the expression level of IL-6 and TNF-α in DM-CON group as compared to diabetic sham ones (P<0.001). Nevertheless, the levels of IL-6 and TNF-α were suppressed by RLIPost treatment (P<0.05 or P<0.001 vs.DM-CON).

### Lung histopathological changes

**Fig 5:**
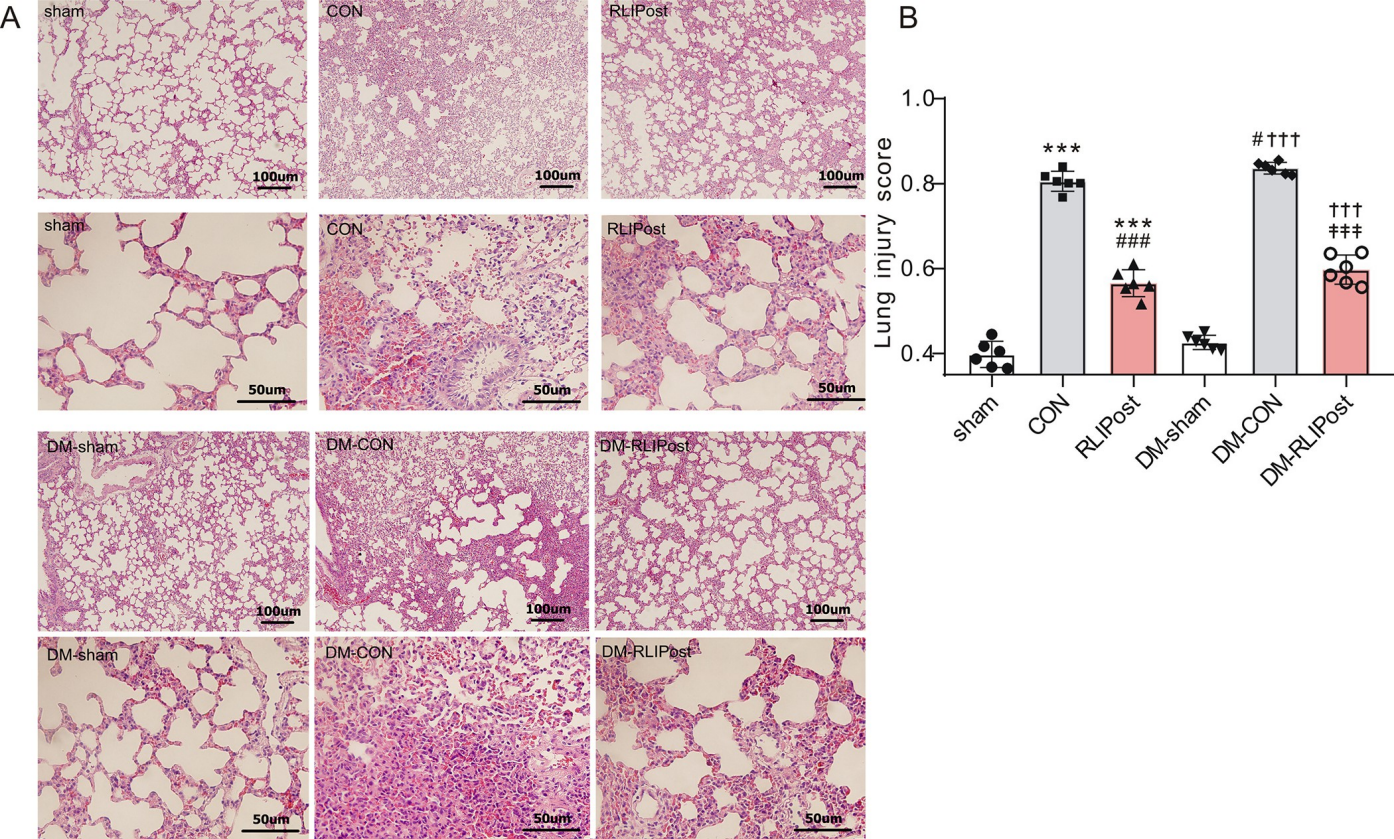
RLIPost preserves pulmonary histological structures post I/R. A. Representative photographs showing H&E-stained lung sections post I/R. sham: sham-operated, CON: control, RLIPost: remote liver ischemia postconditioning, DM: STZ-induced diabetes. B. Histological evaluation of pulmonary damage after reperfusion in experimental groups. n = 4–6 in each group. The severity of lung damage was graded (see the ‘Methods‘ section). Values are mean ± SD. ***P<0.001, versus the sham group, ^#^P<0.05, ^###^P<0.001 versus the CON group, ^†††^P<0.001 versus DM-sham, ^‡‡‡^: P<0.001 vs. DM-CON.

Hematoxylin and eosin staining was used to evaluate the histopathological changes in the left upper lobe of lung tissues. Representative images of lung sections are shown in ***Fig 5A***. The pulmonary damage was also quantified using a histologic scoring system as we previously described [16]. The lung tissues taken from the sham-operated rats exhibited almost normal histological structure with scattered neutrophil infiltration. Histological examination in diabetic or non-diabetic lung tissues exposed to I/R injury showed severe pulmonary damage (P<0.001, CON vs. sham and P<0.001, DM-CON vs. DM-sham), i.e., extensive neutrophil infiltration, alveolar hemorrhage, inter-alveolar septum thickening, and pulmonary edema. However, the degree of I/R-induced alveolar damage was markedly attenuated upon RLIPost treatment, as evidenced by reduced structural damage and lower lung injury scores (P<0.001, RLIPost versus CON, P<0.001, DM-RLIPost versus DM-CON ***Fig 5B***).

### RLIPost inhibited I/R induced pulmonary apoptosis

**Fig 6:**
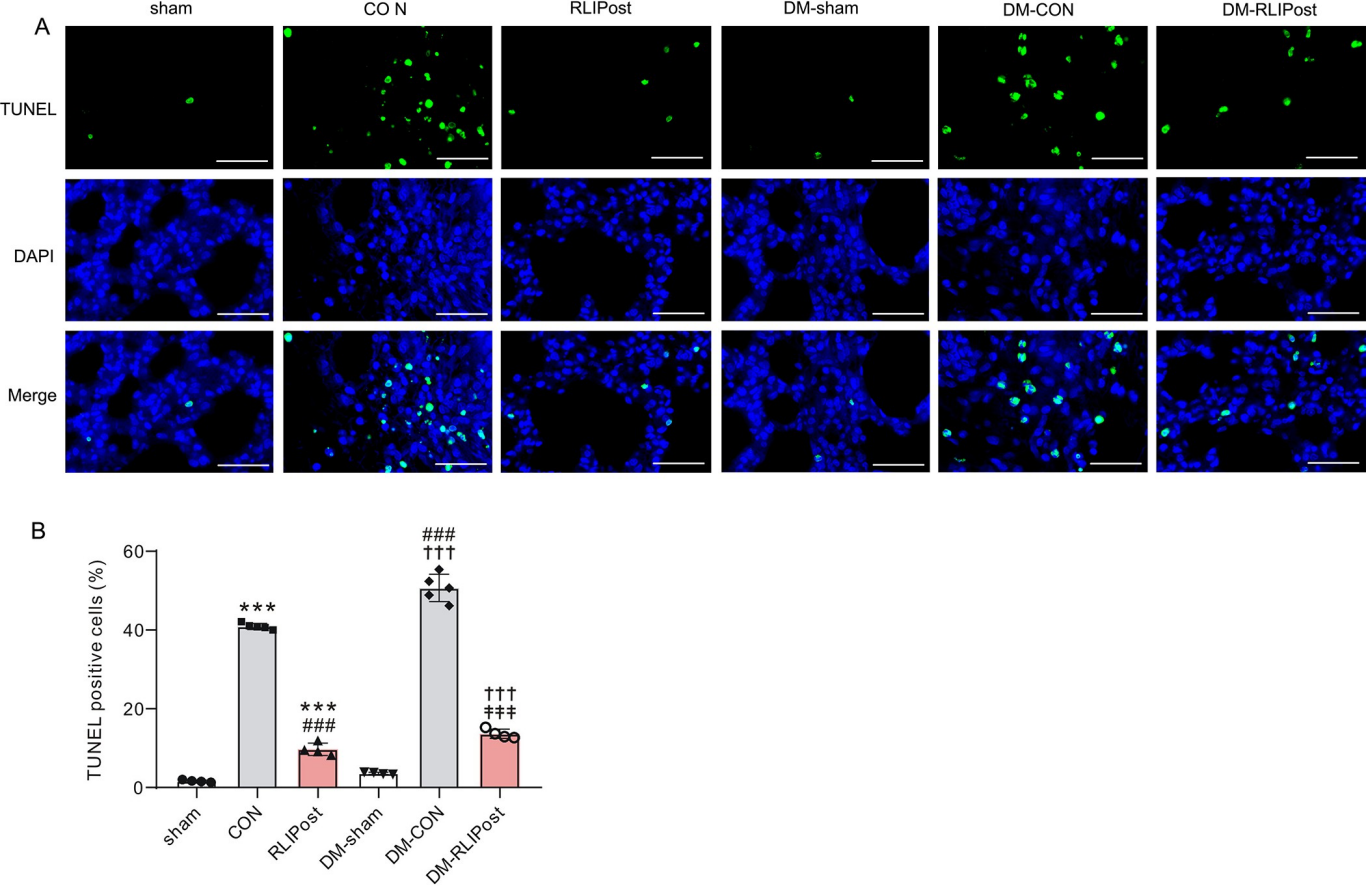
RLIPost inhibited pulmonary apoptosis post I/R. A. Representative TUNEL-stained lung sections of rats subjected to pulmonary I/R injury. TUNEL-positive cells were stained green and nuclei were stained red. Scale bars, 50 μm. sham: sham-operated, CON: control, RLIPost: remote liver ischemia postconditioning, DM: STZ-induced diabetes. B. Bar graph showing the percentage of TUNEL positive-stained cells in the lung sections. Values are mean ± SD. n = 4–5 per group. ***P<0.001, versus the sham group, ^###^P<0.001 versus the CON group, ^†††^P<0.001 versus DM-sham, ^‡‡‡^: P<0.001 vs. DM-CON.

Lung tissue apoptosis was determined by the terminal deoxynucleotidyl transferase dUTP nick end labeling (TUNEL) assay. As shown in ***Fig 6A and 6B***, TUNEL-positive nuclei were stained green. Rats in control and RLIPost groups presented more apoptotic nuclei than in the sham-operated group (all P<0.001 vs. sham). In contrast, as compared with the non-RLIPost-treated control group (40.9±0.8%), the number of TUNEL-positive stained nuclei in the RLIPost group (9.7±1.6%) was significantly reduced (P<0.001), indicating that RLIPost effectively inhibited I/R-induced pulmonary apoptosis. For diabetic rats, the apoptosis of lung cells was up-regulated in the DM-CON group (P<0.001 vs.DM-sham group), which suggested that the I/R injury could promote apoptosis in the diabetic lungs. However, RLIPost treatment effectively attenuated pulmonary apoptosis after reperfusion when compared with the DM-CON group (P<0.001).

### Pulmonary STAT-3 phosphorylation status post I/R

**Fig 7:**
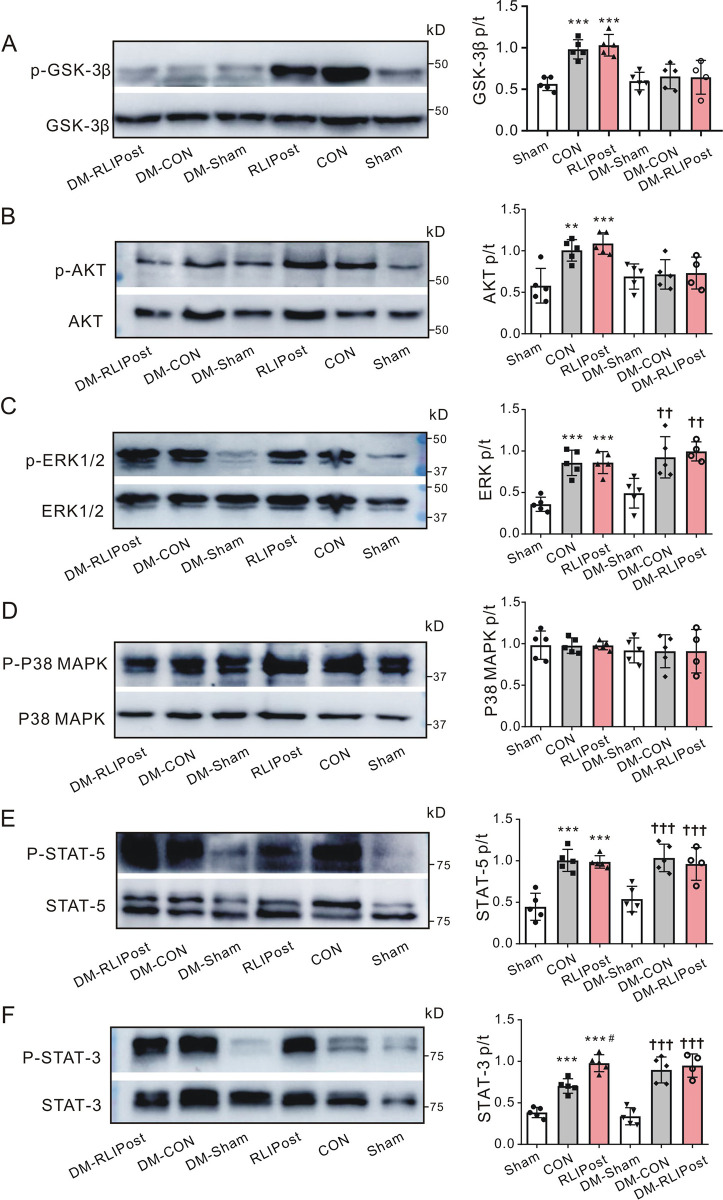
The effect of RLIPost on pulmonary STAT-3 phosphorylation post I/R. ***Left*,** Representative Western blots of phospho-(p) GSK-3β and total (t) GSK-3β (**A**), phospho-(p) AKT and total (t) AKT (**B**), phospho-(p) ERK1/2 and total (t) ERK1/2 (**C**), phospho-(p) P38-MAPK and total (t) P38-MAPK (**D**), phospho-(p) STAT-5 and total (t) STAT-5 (**E**) and phospho-(p) STAT-3 and total (t) STAT-3 (**F**) in rat lungs after I/R injury. sham: sham-operated, CON: control, RLIPost: remote liver ischemia postconditioning, DM: STZ-induced diabetes. ***Right*,** quantification of the p-GSK-3β/t-GSK-3β in (A), p-AKT/t-AKT in (B), p-ERK1/2/t-ERK1/2 in (C), p-P38-MAPK/tP38-MAPK in (D), p-STAT-5/t-STAT-5 in (E), and p-STAT-3/t-STAT-3 in (F). **P<0.01, ***P<0.001, compared with sham-operated rats, ^#^P<0.05, compared with control rats, ^††^P<0.01, ^†††^P<0.001 versus DM-sham post I/R (by One-way ANOVA). n = 4–5 per group.

We previously demonstrated that remote liver ischemic preconditioning protected heart [6] and lung [10] against I/R injury by activating pro-survival signaling cascades. To further elucidate the protective role of RLIPost in non-diabetic and diabetic lungs post-I/R, we determined levels of pro-survival signaling molecules in different signaling pathways known to be engaged in lungs in response to ischemia/reperfusion injury in all groups after 2 hours of reperfusion, specifically phosphorylated glycogen synthase kinase-3β (GSK-3β), protein kinase B (AKT), extracellular signal-regulated kinase 1/2 (ERK1/2), mitogen-activated protein kinase p38 (p38 MAPK), the signal transducer and activator of transcription 3 (STAT3) and the signal transducer and activator of transcription 5 (STAT5). We found that the total protein levels of the abovementioned proteins were not different among groups (P>0.05). Although I/R stimuli caused significant increases in GSK-3β (P<0.001, ***Fig 7A***), AKT (P<0.01, ***Fig 7B***) and ERK1/2 (P<0.001, ***Fig 7C***) phosphorylation in non-diabetic control lungs vs. non-diabetic sham-operated lungs, remote liver postconditioning did not further improve their phosphorylation levels in non-diabetic lungs (all P>0.05 vs. CON). The phosphorylation level of P38 MAPK was similar among three non-diabetic experimental groups (P>0.05, ***Fig 7D***), all indicating that GSK-3β/AKT/ERK1/2/P38 MAPK related pathways were not associated with RLIPost-induced pulmonary protection in non-diabetic lungs. Meanwhile, we did not detect differences in phosphorylation levels of GSK-3β (P>0.05), AKT (P>0.05), ERK1/2 (P>0.05) or p38 MAPK (P>0.05) proteins between diabetic control and diabetic RLIPost groups. We previously found that remote liver preconditioning ameliorated pulmonary damage via activating STAT-3 in the survivor activating factor enhancement (SAFE) signaling pathway [10]. In the current study, we tested STAT-3 and STAT-5 in the SAFE pathway and found that the ratio of phosphorylated to total pulmonary STAT-5 protein was similar between non-diabetic control and non-diabetic RLIPost groups post I/R (P>0.05, ***Fig 7E***). In contrast, there were marked increases in phosphorylated STAT-3 immunoreactivity in the control group compared with those in the sham-operated group (P<0.001), and the phosphorylation levels of STAT-3 were further increased in the postconditioned lungs relative to that in the control lungs (P<0.05, ***Fig 7F***), suggesting that STAT-3 may be responsible for RLIPost-related pulmonary protection against I/R injury in non-diabetic lungs. However, rats in the DM-CON and DM-RLIPost groups exhibited similar increases in phosphorylation of pulmonary STAT-5 (P>0.05 vs. DM-CON) and STAT-3 (P>0.05 vs. DM-CON) post I/R, suggesting that the underlying mechanism for RLIPost-induced pulmonary protection may be different in non-diabetic (STAT-3 dependent) versus diabetic (STAT-3 independent) rats.

## Discussion

In this study, we investigated the beneficial effect of remote liver ischemic postconditioning against ischemia/reperfusion-induced pulmonary injury in non-diabetic and diabetic rats. Our data demonstrate that RLIPost protects both non-diabetic and diabetic lungs, as illustrated by the significant improvement in pulmonary function and reductions in pulmonary edema, inflammatory response, structural damage and apoptosis post I/R. Moreover, our data also suggest that RLIPost-induced pulmonary protection is associated with STAT-3-dependent cell-survival signaling pathway in non-diabetic but not diabetic rats.

Remote ischemic preconditioning (RIPC) and postconditioning (RIPost) are novel and promising therapeutic approaches whereby brief cycles of ischemia and reperfusion stimuli conducted in a remote organ (not the target organ) protect the target organ against subsequent sustained I/R injury. A number of preclinical studies have demonstrated the significant cardioprotective effects of RIPC or RIPost against myocardial I/R injury [18,19]. In addition to providing convincing evidence of its critical role in cardioprotection, several reports also showed that RIPC could attenuate acute lung injury after lung resection [20], or confer lung protection due to liver [21] or limb I/R [8]. Furthermore, although the literature on this topic is quite scarce, it has been shown that conditioning stimuli conducted on the limbs can ameliorate lung damage in the setting of lung I/R injury [22]. Unfortunately, in comparison with the limbs or other organs, little is known regarding the effectiveness of ischemic conditioning performed in the liver, the largest metabolic organ in the body. We and others have previously found that remote liver ischemic preconditioning could effectively protect hearts against myocardial I/R injury in different animal models [23,24]. Importantly, using a rat model of pulmonary I/R injury, we further demonstrated the existence of remote liver preconditioning-induced pulmonary protection [10]. Using different experimental techniques and a range of indicators, our current study extends these findings by showing that remote liver postconditioning ameliorates pulmonary damage post-reperfusion insult in rats in the presence or absence with diabetes.

Pulmonary edema is a common consequence of acute lung injury. The lung wet-to-dry (W/D) weight ratio reflected its water content and was calculated as an indicator of lung edema. We found in our current study that RLIPost markedly reduced W/D ratios and prevented the development of pulmonary edema post I/R. There is an increasing body of evidence indicating that the pro-inflammatory cytokines, such as IL-6 and TNF-α, are vital mediators in the pathogenesis of I/R injury and the release of these cytokines is required to produce the damage [25]. We found that RLIPost significantly attenuated lung inflammation and reduced the pulmonary expression of pro-inflammatory cytokines. These findings suggest that RLIPost attenuates acute lung damage induced by I/R injury via an anti-inflammatory mechanism. In addition, RLIPost also increased pulmonary oxygenation, suppressed apoptosis, and preserved the lung histological structure. These findings all highlight the protective role of RLIPost during the pulmonary I/R process.

Increasing evidence has shown that the lung is a target organ in patients with either type 1 or type 2 DM [26]. Diabetic hyperglycemia damages the respiratory system and causes several physiological and structural abnormalities of pulmonary function [27]. Therefore, respiratory disorders have been frequently observed in diabetic patients [28]. Notably, it has been shown that DM increases pulmonary susceptibility to I/R injury [29]. It is well established that the efficacy of ischemic conditioning-induced cardioprotection can be disrupted in the diabetic heart [15]. Previously, it was not known whether diabetes would blunt the pulmonary protective effect offered by ischemic conditioning, especially remote ischemic conditioning. Indirect evidence from Neto *et al*. showed that ischemic preconditioning protected diabetic lungs submitted to intestinal or hepatic reperfusion injury [30]. The current results extend these previous findings, demonstrating that after treatment with RLIPost, the degree of lung edema, pulmonary histological damage, inflammatory cytokines, and cellular apoptosis are markedly attenuated in diabetic-ischemic lungs, indicating a direct protective role of RLIPost against pulmonary I/R injury in diabetes.

The reperfusion injury salvage kinase (RISK) pathway and the survivor activating factor enhancement (SAFE) signaling pathway are two of the most important signaling pathways associated with I/R injury. The RISK pathway was first proposed by Yellon’s group in 2002 [31]. Several protein kinases are vital components of the RISK pathway, including ERK1/2, AKT, P38 MAPK, and their downstream signaling molecule GSK-3β. It has been well characterized that RISK serves as a common pathway for organ protection against I/R injury. It can be activated by pharmacological agents or endogenous stimulation given either prior to ischemia or at the onset of myocardial reperfusion. The link between ischemic preconditioning- or postconditioning-related cardioprotection and the RISK pathway has been established. We and others have previously demonstrated that the ischemic conditioning-mediated infarct sparing effect is associated with RISK-dependent signaling pathways [24,32].

We previously found that remote liver ischemic conditioning conducted prior to pulmonary ischemia protected lungs against pulmonary I/R injury in a RISK-independent manner [10]. In the current study, we further investigated the protective role of RLIPost and similarly, we found that in both diabetic rats and non-diabetic rats, RLIPost-induced pulmonary protection was independent of RISK activation. These results suggest that there may be an alternative signaling pathway responsible for the mediation of RLIPost compared to preconditioning protection.

Signal transducers and activators of transcription (STAT) factors, the key components in the SAFE pathway, function as modulators of signaling and sensors in response to stimuli and cellular stress. STATs can be activated by phosphorylation. It has been shown that activation of the STAT-3 pathways promotes cardiac myocyte survival and inhibits apoptosis after myocardial I/R injury [33]. Moreover, following I/R injury, STAT-3 deficient mice were previously shown to be more susceptible to cardiac injury and exhibited larger infarct size with increased cardiac apoptosis when compared to their wild-type littermates [34]. Meanwhile, Smith *et al*. also found that the beneficial effect of ischemic preconditioning was absent in STAT-3 deficient mice [35]. Taken together, these studies revealed that STAT-3 may serve as an anti-apoptotic signaling factor, increasing cell survival after an ischemic insult. Interestingly, we previously found that in a rat model of pulmonary I/R injury, pulmonary I/R caused STAT-3 phosphorylation, and remote liver ischemic preconditioning further enhanced STAT-3 activation (via phosphorylation) [10]. Consistent with our previous study, here we found a similar pattern for STAT-3 activation upon remote liver postconditioning treatment in the non-diabetic rat lungs, indicating that liver ischemic preconditioning and postconditioning may share similar molecular mechanisms in the pulmonary protective process after I/R injury. Notably, we also observed that RLIPost exerted potent lung protection against pulmonary I/R injury in diabetic lungs, however, RLIPost induced similar pulmonary STAT-3 protein phosphorylation in diabetic lungs to that observed in diabetic controls. Similar to the situation for STAT-3, we also did not observe statistically significant differences in the phosphorylation level of other candidate protective pathway proteins in RLIPost versus control lungs in diabetic rats. It is possible that other pulmonary-protective pathways not explored in this study are induced by RLIPost after pulmonary I/R in STZ-induced diabetic models. It will be of interest in the future to determine the exact underlying signaling mechanisms.

We acknowledge several limitations of this study. First, in order to produce type 1 diabetes mellitus (T1DM), we used streptozotocin (STZ), which induces pancreatic islet β-cell destruction. This animal model does not incorporate the long-term inflammation and pathological remodeling required for the development of type 2 DM. Therefore, our study may not be representative of all diabetic statuses. However, the current results reveal the protective role of remote ischemic postconditioning during pulmonary I/R injury under acute hyperglycemic circumstances. Second, based on our study, STAT-3 is a candidate signaling molecule associated with RLIPost-induced pulmonary protection in non-diabetic lungs. However, pharmacological or genetic interventions may be required for further validation, and it is conceivable that there are additional signaling cascades involved in the pathophysiology. Meanwhile, the reason why RLIPost did not alter the phosphorylation level of STAT-3 post I/R in diabetic lungs remains unclear. Using the current experimental protocol and study design, we were unable to elucidate the exact molecular mechanisms responsible for the RLIPost-related therapeutic properties in diabetic lungs. Third, we only studied two inflammatory cytokines (TNF-α and IL-6) in the current study; therefore, the possibility that RLIPost may alter the extent of expression of other pro-inflammatory cytokines post I/R has not been ruled out.

In conclusion, the present study demonstrates that remote liver ischemic postconditioning protects non-diabetic and diabetic lungs against pulmonary ischemia and reperfusion injury, as assessed by reduction in pulmonary edema, cell apoptosis, inflammatory responses and restoration of pulmonary function and maintenance of normal alveolar structure. This liver postconditioning-associated pulmonary protection is exerted, at least in part, by the activation of STAT-3-dependent signaling pathway in non-diabetic lungs, but not in diabetic lungs.

## Supporting information

S1 File (PDF)
