# Supplementary material for: Protective effect of remote liver ischemic postconditioning on pulmonary ischemia and reperfusion injury in diabetic and non-diabetic rats
Source: PLoS One. 2022 May 26;17(5):e0268571. doi: 10.1371/journal.pone.0268571 (PMC9135201; doi:10.1371/journal.pone.0268571)
Supplement: S1 File — (PDF) [file pone.0268571.s001.pdf]

All proteins tested in our current study were phosphorylated proteins. The phosphorylated and total protein are on the same location with same molecular weight in the same membrane. Tissue samples were homogenized and centrifuged. Protein concentration was determined by BCA method. After detecting the expression of phosphorylated protein, the proteins in the membrane were stripped with stripping buffer. The membrane was re-probed with the total protein at the same molecular weight in the same membrane. The phosphorylation levels of each protein were normalized with their respective total proteins.

Figure 7A:

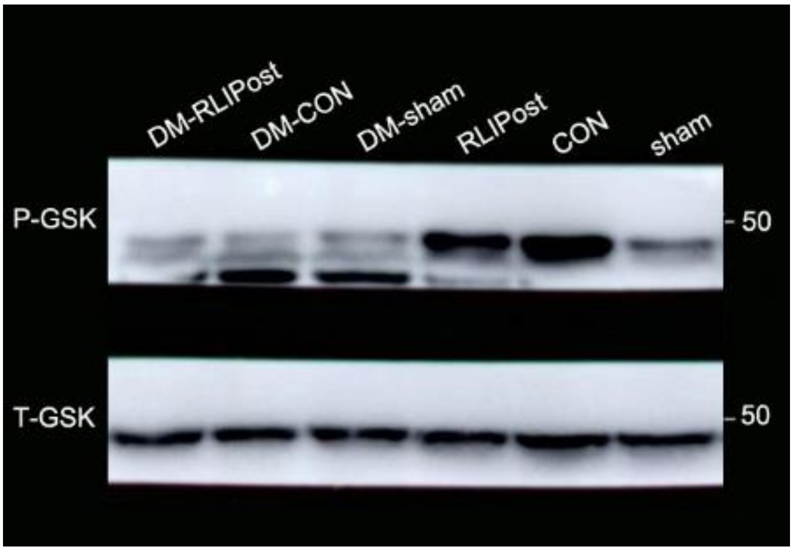

Figure 7B:

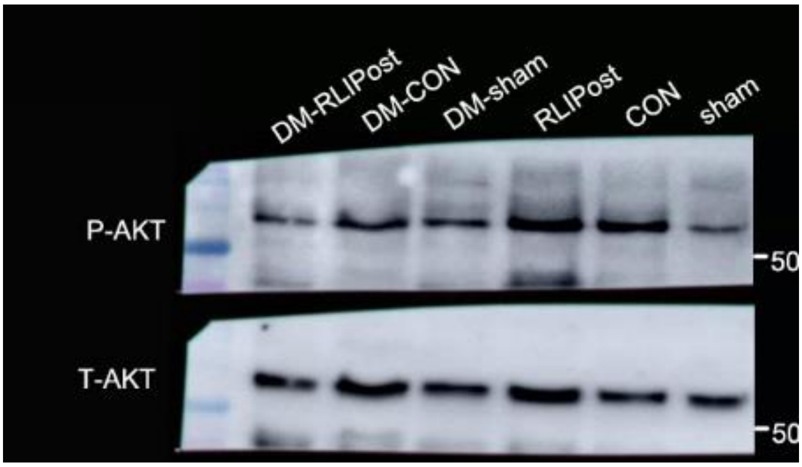

Figure 7C:

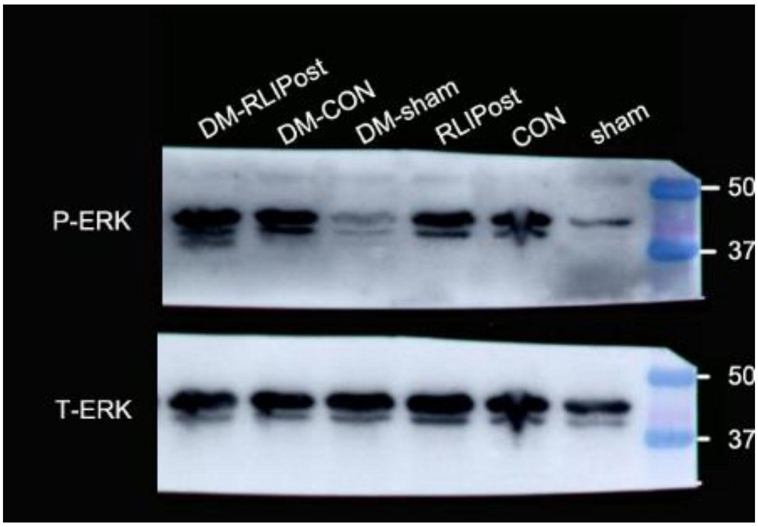

Figure 7D:

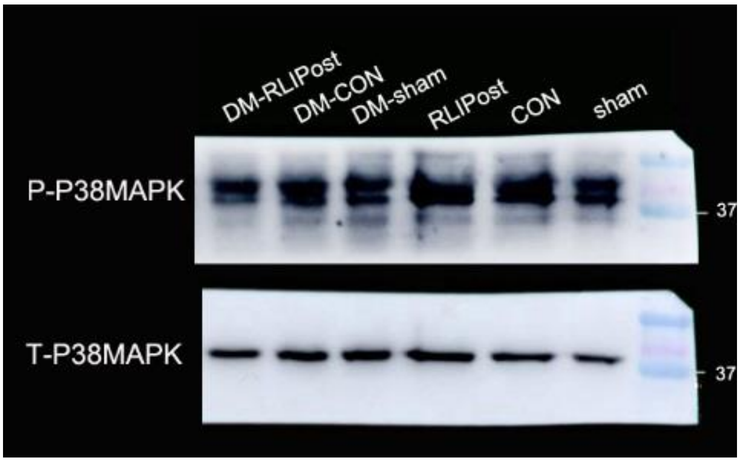

Figure 7E:

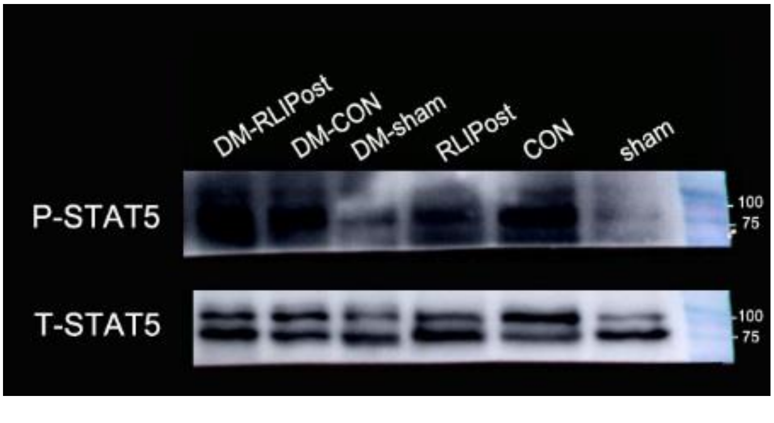

Figure 7F:

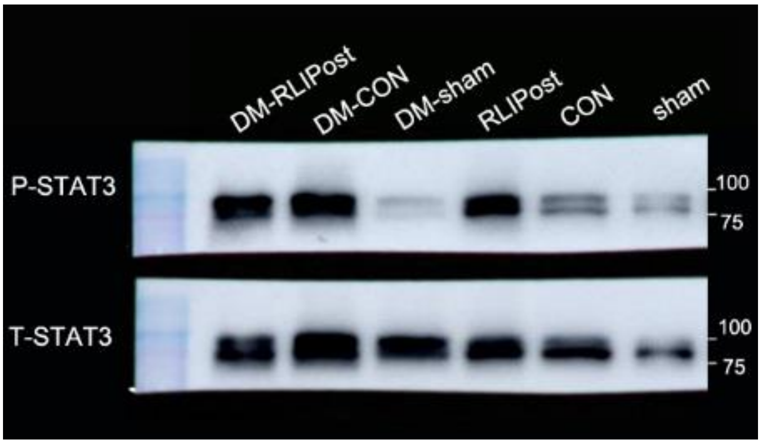
DM-RLIPost
DM-CON
DM-sham
RLIPost
CON
sham
P-STAT3
100
75
T-STAT3
100
75
